# c-Met as a Prognostic Marker in Gastric Cancer: A Systematic Review and Meta-Analysis

**DOI:** 10.1371/journal.pone.0079137

**Published:** 2013-11-04

**Authors:** Shan Yu, Yiyi Yu, Naiqing Zhao, Jianlan Cui, Wei Li, Tianshu Liu

**Affiliations:** 1 Department of Medical Oncology, Zhongshan Hospital, Fudan University, Shanghai, People’s Republic of China; 2 Department of Biostatistics, Fudan University, Shanghai, People’s Republic of China; University of Torino, Italy

## Abstract

**Background:**

c-Met has been recognized as an important therapeutic target in gastric cancer, but the prognostic property of the c-Met status is still unclear. We aimed to characterize the prognostic effect of c-Met by systematic review and meta-analysis.

**Methods:**

We identified 15 studies assessing survival in gastric cancer by c-Met status. Effect measure of interest was hazard ratio (HR) for survival. Meta-regression was performed to estimate the relationship between HR and disease stage. Random-effects meta-analyses were used to account for heterogeneity.

**Results:**

15 eligible studies provided outcome data stratified by c-Met status in 2210 patients. Meta-analysis of the HRs indicated a significantly poorer Os in patients with high c-Met expression (average HR=2.112, 95%CI: 1.622–2.748). Subgroup analysis showed the prognostic effect of c-Met was identical in protein-level and gene-level based methodology. The same effect was also seen in Asian and Western ethnicity subgroup analysis. Meta-regression showed HR was not associated with disease stage.

**Conclusions:**

Patients with tumors that harbor high c-Met expression are more likely to have a worse Os, with this prognostic effect independent of disease stage. c-Met status should be evaluated in clinical prognosis.

## Introduction

Gastric cancer(GC) is one of the most common human malignant diseases and remains the second leading cause of cancer-related death worldwide[1]. Although recent diagnostic and therapeutic advances have improved the clinical outcomes of patients with early stage of gastric cancer, the prognosis for advanced stage remains extremely poor, and overall 5-year survival rates are approximate 15%[2,3], so there is a continuous need for understanding the mechanisms underlying gastric cancer as well as identifying new molecular targets for treatment. To date, oncologists have identified several factors that promote the development of gastric cancer for therapeutic targeting, and prominent amongst these is c-Met.

The receptor tyrosine kinase c-Met is encoded by MET oncogene. This receptor and its hepatocyte growth factor (HGF) ligand have been found frequently dysregulated in gastric carcinomas[4-7]. c-Met is overexpressed in approximately 20% of gastric cancer cell lines[8] and 18–82% of gastric cancer cases[7,9-12]. The c-Met/HGF pathway stimulates the proliferation, invasion, angiogenesis as well as protection from apoptosis in cancer cells[13,14]. Therefore, c-Met has been recognized as an important therapeutic target in antineoplastic strategies, and also has shown to possess predictive properties for the treatment with the monoclonal antibody to HGF in locally advanced or metastatic gastric cancer[15]. 

However, when it comes to the prognostic properties of the c-Met status, there still seems to be no consensus, despite a relatively large number of studies on c-Met in gastric cancer have looked into the association between a c-Met-positive status and survival. In this current systematic review we have utilized the already existing literature to address the issue on the prognostic properties of a c-Met-positive status in gastric cancer.

## Materials and Methods

### 1. Publication search

Systematic computerised searches were performed using the electronic database Pubmed(up to 30th May 2013). The search strategy used the keywords ‘c-Met’, or ‘MET’, and ‘gastric cancer’, or ‘gastric carcinoma’ or ‘stomach neoplasm’. English language published studies were eligible if they met the following criteria: (1) patients had a diagnosis of gastric cancer; (2) overall survival(OS) or progression-free survival(PFS) were analyzed stratified by c-Met status; (3) the results were part of an original analysis; (4) when the same patient population was used in several publications, only the most recent, largest or complete study was included in the meta-analysis. Data from review articles, abstracts, and letters were not included.

### 2. Data extraction

Study characteristics were extracted from the eligible articles and summarized in a consistent manner to aid comparison. The following data were collected from each study: first author’s name, year of publication, number of patients screened, disease stage, clinical treatment, methodology of c-Met analysis including the threshold used to dichotomize c-Met as ‘high’ and ‘low’, progression-free survival and overall survival stratified by c-Met status and hazard ratio(HR) with 95% confidence intervals(CI) for PFS or OS. When HRs and its confidence intervals were not directly reported, they were estimated from other data, such as number of patients in each group and Kaplan–Meier curves for overall survival, using the published methodology[16]. Both unadjusted and adjusted HR estimates were sought for each study. When relevant effect estimates were not obtainable using the methods above, the study was excluded from the meta-analysis. 

### 3. Statistical analysis

The endpoints were PFS and OS. The association between c-Met level and PFS or OS was evaluated using the hazard ratio of high c-Met level patients over low c-Met level patients and 95% confidence interval from univariate and multivariate Cox proportional hazards models, so a HR of 1 indicates a lack of association between c-Met level and risk of death, a HR of greater than 1 indicates a greater risk of death in high c-Met level patients, and a HR less than 1 indicates a greater risk of death in low c-Met level patients.

A fixed effects model was initially used to calculate the pooled HR estimates. If the I^2^ statistic was more than 50% or the fixed effects p value for the I^2^ statistic was less than 0.10, indicating significant heterogeneity across studies, a random effects model was then used for calculating the pooled estimate. 

To establish the effect of methodology heterogeneity among studies on meta-analyses conclusions, subgroup analyses were conducted by study designs. In the subgroup analysis of c-Met expression ascertainment method, studies were classified as either protein-level method subgroup which performed immunohistochemistry(IHC) to stratify c-Met expression or gene-level method subgroup, such as reverse transcriptase quantitative polymerase chain reaction(RT-qPCR), southern blot, and fluorescence in situ hybridization(FISH), which dichotomize c-Met status by gene amplification, as reported in the given publication. Clinical heterogeneity was established by the subgroup analysis of ethnicity, in which studies were classified as Asian or Western subgroup. To demonstrate whether HR was associated with the disease stage of the GC patients, we performed the meta-regression analysis of logarithm transformed outcomes(HR) against percentage of advanced stage patients in each study, and HR was considered to be associated with disease stage when the p value of the model was less than 0.05. 

Potential publication bias were assessed by performing Egger’s test(p<0.05 was considered representative of statistically significant publication bias) for meta-analysis including 10 or more studies. All statistical analyses were performed using STATA version 11.0.

## Results

### 1. Eligible Studies and Studies characteristics

**Figure 1:**
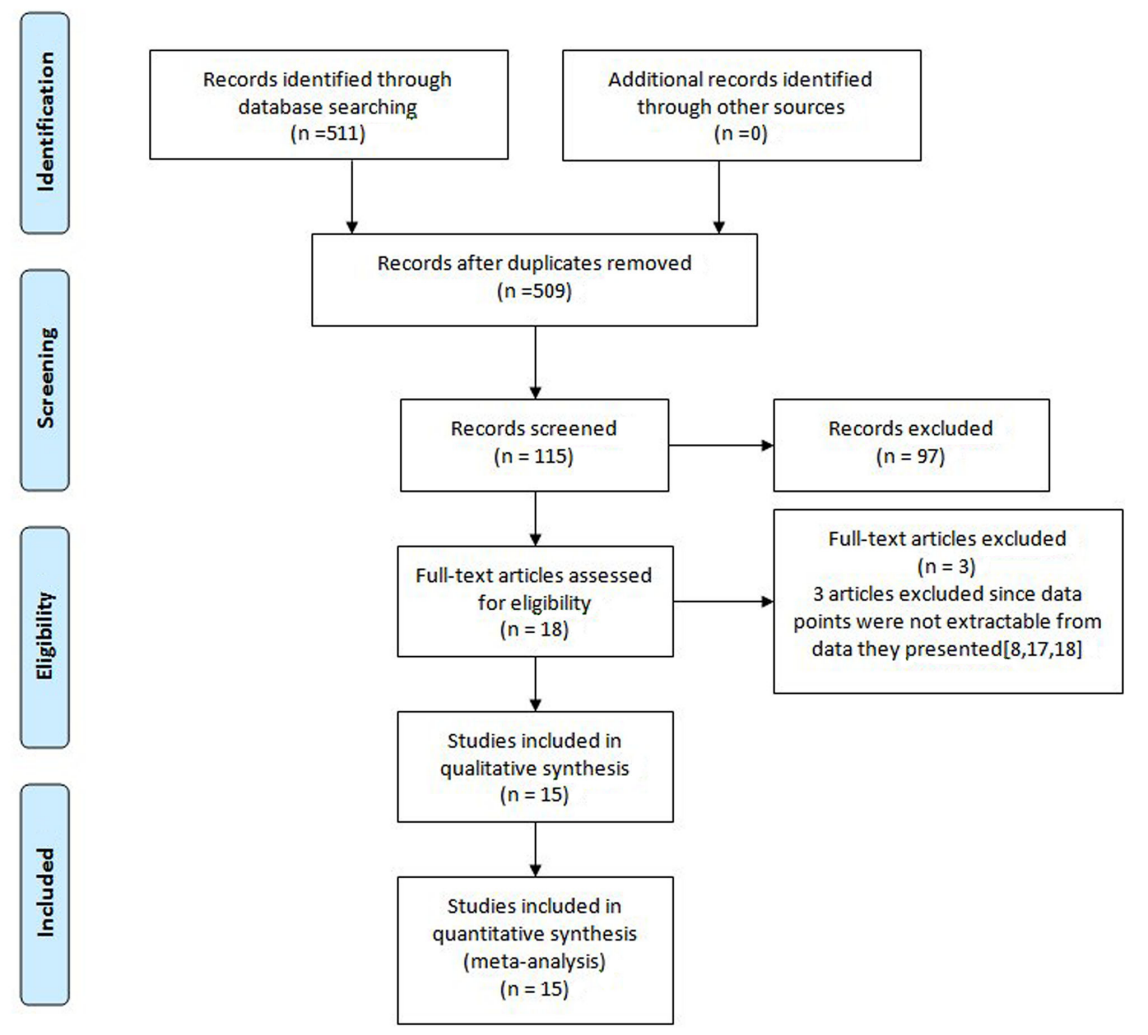
PRISMA flow Chart of selection process to identify eligible studies. doi:10.1371/journal.pmed 1000097.

The flowchart of our study is shown in Figure 1. From 511 abstracts we found, 493 were excluded based on our inclusion criteria. Among the 18 articles that were left for eligibility assessment, 3 articles were excluded since data points were not extractable from data they presented[8,17,18]. Finally, we identified 15 eligible studies[4,7,19-31] which provided outcome data stratified by c-Met status from 7 countries, and their characteristics are summarized in Table 1. In two studies [29,30] outcome data were presented separately by c-Met expression ascertainment method, and these datasets were treated separately. Specifically, Lee et al.[29] stratified outcome data into two groups, and one subgroup which used IHC to score c-Met expression was excluded since the relevant effect estimates could not be obtained. Sample sizes of the datasets assessed for overall survival ranged from 35 to 472(median 107; Table 1), with data from a total of 2210 patients available for the meta-analyses. Fourteen of these studies were of retrospective design. One of these studies were of prospective design. Of the 15 studies, 11[4,19,22-24,26-31] included patients with early and advanced disease(stage I-IV), however, none of these studies separated their patients into subgroups. The remaining four studies[7,20,21,25] included patients with stage I-III. All patients in our meta-analysis underwent curative or noncurative primary tumor resection, and none of them received chemotherapy or radiotherapy before the surgery. In five studies[7,22,27-29], patients with advanced stage were given postoperative chemotherapy or chemoradiation, while patients in another study didn’t receive any postoperative treatment[21]. Nine studies[4,19,20,23-26,30,31] didn't mention the postoperative treatment. None of these studies mentioned palliative treatment after relapse except one[20], in which all patients who experienced relapse underwent fluorouracil-based chemotherapy(Table S1). None of the treatment used in these studies was decided by c-Met status. Mean follow-up time data were presented by most investigators, with a median of 71 months(range from 20 to 160 months). OS was reported in all studies, but progression-free survival was only presented in one study[20]. Nine studies[4,19-24,28,29] presented data on HR, with 95% CI for OS directly. One study[30] presented HR without 95% CI for OS. HRs and 95%CIs were not directly presented in the remaining five studies and they were estimated from Kaplan–Meier curves. Adjusted results were provided in eight studies[4,19-24,28] while unadjusted results were provided in only two studies[29,30].

**Table 1:** Summary of studies reporting c-Met expression and outcomes in gastric cancer patients.

Study (first author +publication date)	No. of patients^a^	Country	Disease Stage	Percentage of advanced stage(%)	Method to stratify c-Met status	high c-Met expression(%)	HR	95%CI
Toiyama 2011	100	Japan	I-IV	34.1	RT-qPCR	24	2.99*	1.67-5.35*
Catenacci 2011	36	USA	I-IV	NS	RT-qPCR	8	4.7	1.31-16.9*
Catenacci 2011	35	USA	I-IV	NS	IHC	43	1.34	0.93-1.92*
Lee 2012	438	Korea	I-IV	10.8	SISH	NS	2.27	1.05-4.93
Li 2012	114	China	I-IV	64.9	IHC	82	0.578	0.221-1.513
Taniguchi 1998	102	Japan	I-IV	NS	IHC	42	1.9*	0.74-4.85*
Tsugawa 1998	70	Japan	I-IV	44.4	Southern Blot	10	9.3*	3.18-27.25*
Nakajima 1999	128	Japan	I-III	NS	IHC	46	1.62*	0.53-4.90*
Huang 2001	45	Taiwan	I-IV	64.4	IHC	71	9.3	1.22–70.81
KUBICKA 2002	42	Germany	I-III	NS	IHC	26	3.77*	1.54-9.24*
Han 2005	50	Korea	I-IV	20.0	IHC	NS	0.651	0.053-1.493
DREBBER 2008	112	Germany	I-IV	31.6	IHC	73	1.9	1.0-3.5
Lee 2011	472	Korea	I-IV	22.4	RT-qPCR	21	1.601	1.078-2.380
Zhao 2011	136	China	I-III	NS	IHC	74	1.879	1.089-3.241
Graziano 2011	216	Italy	II-III	NS	FISH	10	2.91	1.65-5.11
Shi 2012	114	China	I-IV	4.7	RT-qPCR	29	2.1	1.20-3.69

RTqPCR, reverse transcriptase quantitative polymerase chain reaction; IHC, immunohistochemistry; SISH, silver in-situ hybridization; FISH, fluorescence in situ hybridization; NS, not shown;HR, hazard ratio; CI, confidence interval.

^a^ number of patients assessable for c-Met expression and overall survival.

* estimated result from data presented in paper using published methodology.

### 2. c-Met status assignation

c-Met evaluation was performed by IHC in 9 studies [4,7,21,23-25,27,28,30], and RT-qPCR in 4 studies[19,22,30,31]. Another three studies used FISH[20], Southern blot[26], and silver in-situ hybridization(SISH)[29], respectively, as their method to stratify c-Met status. In the 9 studies evaluating c-Met expression by IHC, marked heterogeneity was observed between thresholds used to dichotomise c-Met status. One study[21] derived a composite(H) score by multiplying extent cell-staining score(0–4; 0=none, 1=1-10%, 2=11-50%, 3=51-75%, 4=>75%) by intensity score(0–3; 0=none, 1=weak, 2=moderate, 3=strong) with H-score lower than 3 designated c-Met low. Another study[28] used the same intensity score but a different extent score(0–3; 0=none, 1=<35%, 2=35-75%, 3=>75%) with H-score more than 4 designated c-Met high. In five studies, percentage of cells staining was examined alone, with samples ≥5%[4,7], >10%[25], or >30%[23,27] designated c-Met high. In the remaining two studies, staining intensity grade lower than 2[30] or 3[24] represented low levels of c-Met expression. 

In five studies which assigned c-Met expression at gene level, high c-Met status was defined as the copy of c-Met 7 times[30], 5 times[20], 4 times[19,22] or twice[29] more than that of internal reference. A further study[26] performing Southern blot designated high c-Met status as twice as high as in the normal mucosa. The remaining study[31] used image analyser software to evaluate samples. 

The rate of high c-Met status ranged from 8% to 82% (median, 36%). The observed median proportion of high c-Met expression was 59%(range 26-82%), and 16%(range 8-29%) respectively in studies using IHC and other methods. Two studies did not present the proportion of high c-Met level.

### 3. Main results of overall survival

**Figure 2:**
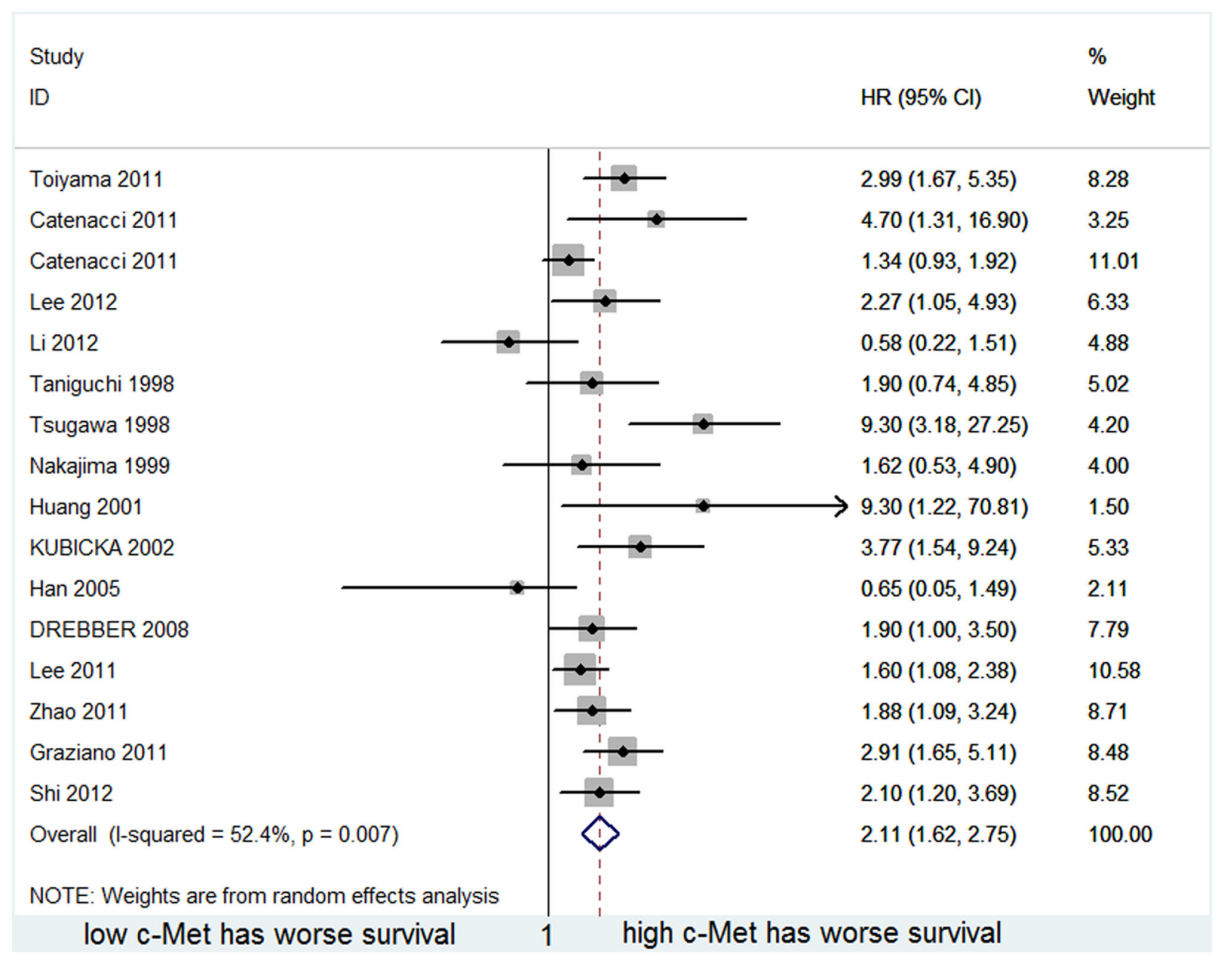
Forest plot showing the meta-analysis of hazard ratio estimates for OS in overall patients.

The results of the meta-analysis of the association between c-Met level and overall survival are provided in Figure 2. 16 datasets were available for pooling OS, 8 with unadjusted[7,25-27,29-31] and 8 with adjusted data points[4,19-24,28]. Meta-analysis of the estimates indicated a significantly poorer OS in patients with high c-Met expression (average HR=2.112, 95%CI: 1.622–2.748). However, the heterogeneity was large(p=0.007, I^2^=52.4%), indicating wide prediction intervals for the prognostic effect in an individual clinical setting. There was no evidence of small study effects using estimates above reported(Egger’s test: p=0.168).

**Figure 3:**
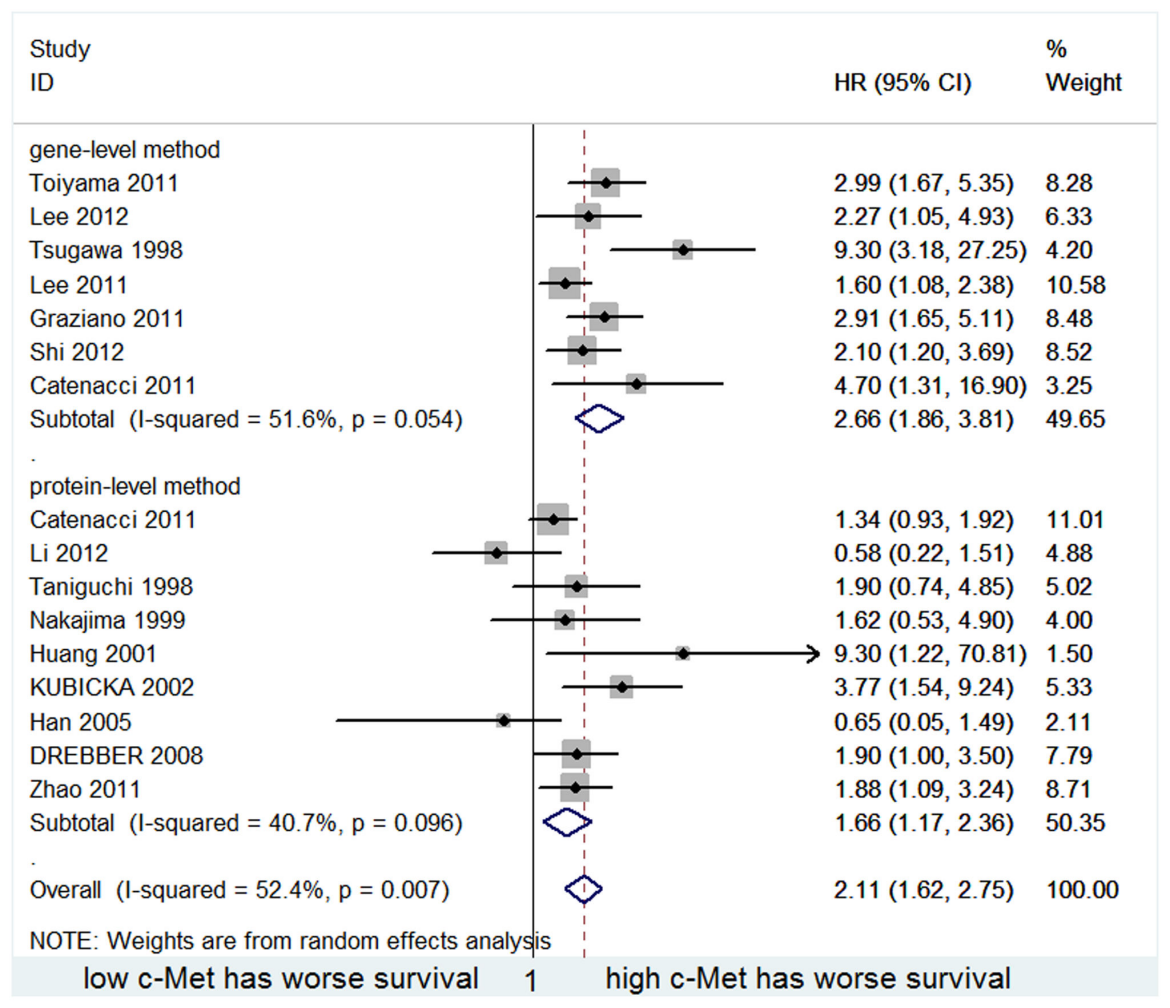
Forest plot showing the meta-analysis of hazard ratio estimates for OS in gene-level subgroup and protein-level subgroup.

The result of the c-Met ascertainment method subgroup analysis is provided in Figure 3. Patients with high c-Met expression levels had a significantly poorer OS in both gene-level method subgroup and protein-level method subgroup (HR=2.661, 95%CI 1.858-3.809 and HR=1.661, 95%CI 1.171-2.357, respectively). There was no statistically significance between the two subgroups(p=0.086). 

**Figure 4:**
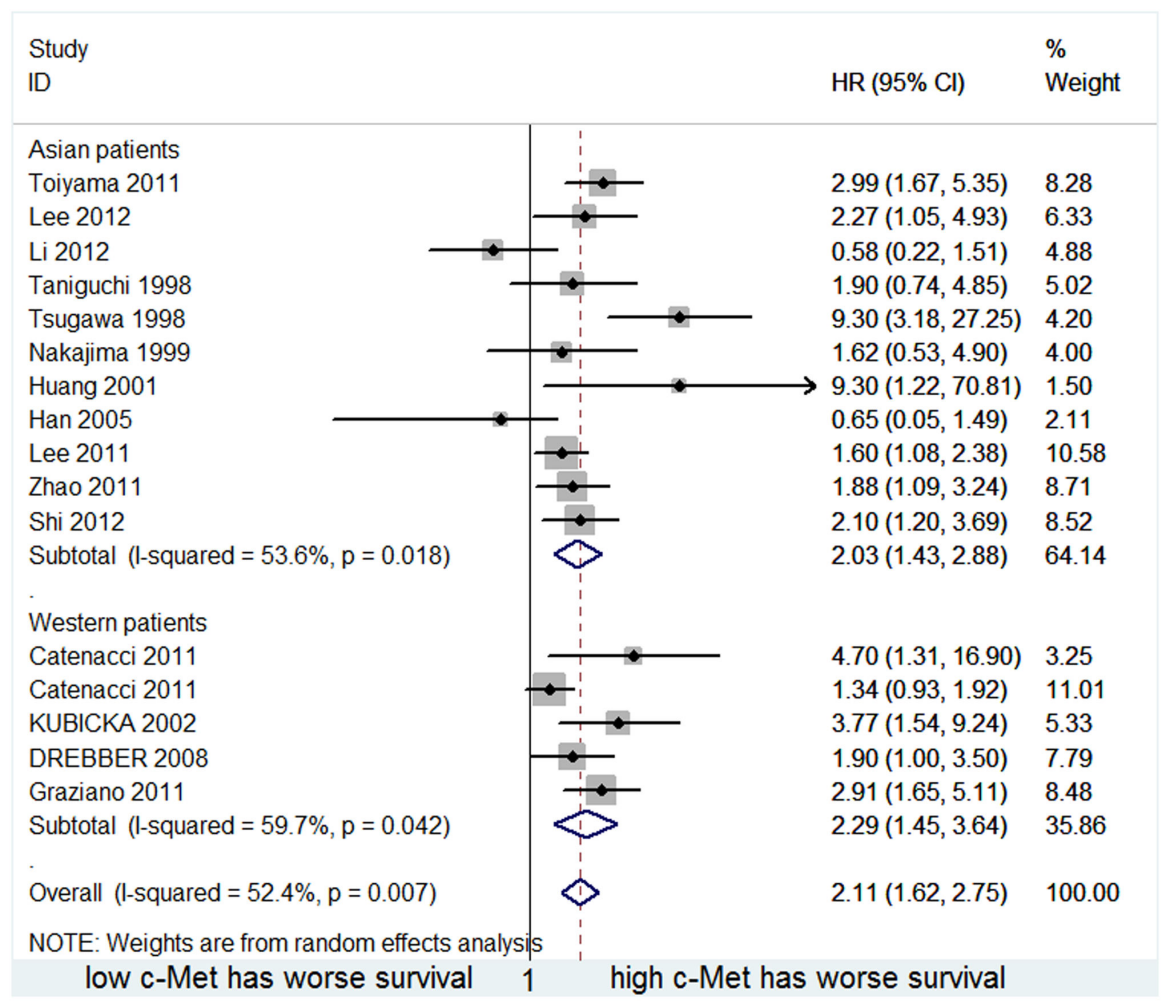
Forest plot showing the meta-analysis of hazard ratio estimates for OS in Asian and Western subgroup.

In an subgroup analysis stratifying patients by ethnicity, there was significant relationship between high c-Met expression and poor OS in both Asian and Western patients(HR=2.032, 95%CI 1.433-2.882 and HR=2.294, 95%CI 1.446-3.640, respectively). The result is provided in Figure 4. No statistically significance was found between the two subgroups(p=0.696).

**Figure 5:**
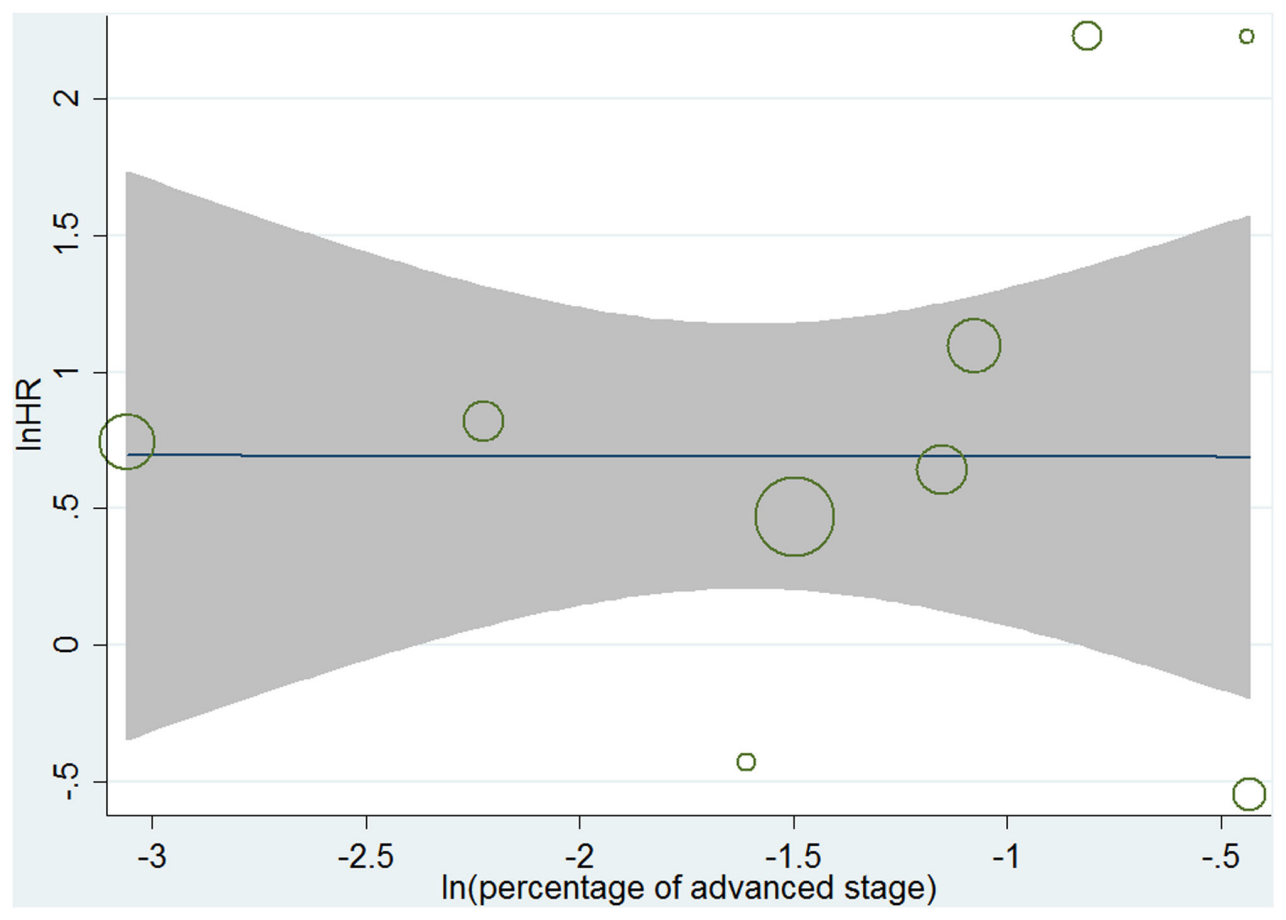
Results of meta-regression. ln(HR)-ln(percentage of advanced stage).

A meta-regression analysis was performed to test the relationship between HR and the disease stage of patients in 9 studies[4,19,22-24,26,28,29,31], among which “advanced stage” standed for stage IV[19,22-24,26,29,31] or stage III and stage IV[4,28]. The overall lnHR had no association with ln(percentage of advanced stage)(p=0.853, Figure 5).

## Discussion

This systematic review has been based on 15 publications covering the period from 1998 to May 2012 and comprises a total of 2210 patients with gastric cancer. The results of this meta-analysis demonstrate the prognostic significance of c-Met expression level in GC patients in Asian and Western countries. In the overall meta-analysis of the association between c-Met level and overall survival, the results indicated a statistically significant increases in overall survival in patients with low c-Met expression in relative to patients with high c-Met expression. 

Subgroup analysis showed that the strength of relationship between c-Met level and overall survival was identical based on the method utilized to ascertain c-Met status. Both gene-level method subgroup and protein-level method subgroup demonstrated statistically significant association between c-Met status and overall survival. In other words, methodology utilized to estimate c-Met status did not effect the prognostic property. However, a statistically significant heterogeneity was seen in both subgroups. The explanation for these rather large variation is likely to be multifactorial such as different populations studied, but the most important aspects are probably the use of varied antibodies for IHC and housekeeping genes for RT-qPCR and the application of different criteria to stratify c-Met status. In a phase II study conducted within the last couple of years which evaluated the effect of HGF antibody and MET pathway biomarkers in advanced GC patients[15], the scoring criteria developed by Kelly et al. identified high c-Met expression as percentage of cells staining≥50% as well as staining intensity grade≥1 for the stained slides, and the total number of MET gene copy>15 for FISH. If this new criteria is widely accepted just as Her2 scoring criteria developed by Hofmann et al.[32] for the ToGA trial, the variation between studies will be minimized. 

Subgroup analysis also showed that both Asian and Western patients harbouring high c-Met level were significantly associated with poorer survival, which did not vary based on ethnicity. While differences in outcomes have been reported between Asian and Western populations with gastric cancer[33,34], our results made c-Met a more widely used prognostic marker.

Disease stage is one of the most important factors that influence OS. In the 15 studies assessed in our meta-analysis, 11 included patients of stage I-IV. However, none of these studies separated their patients into subgroups. With that in mind, we applied meta-regression to detect whether the prognostic property of c-Met would be impacted by the variation of percentage of advanced stage patients among studies. The results showed that the overall HR had no association with the percentage of advanced stage patients in 9 studies, which meant the prognostic effect of c-Met was independent to disease stage. This result further promoted the prognostic value of c-Met in gastric cancer. 

Another important factor for OS is clinical treatment, which includes surgery, postoperative chemotherapy or radiotherapy, and palliative treatment after relapse or disease progression. In our meta-analysis, the pooled HR of five studies in which patients were given postoperative treatment was 1.556, with 95%CI 1.152-2.102(Figure S1), the same as the HR and 95%CI of one study in which patients didn’t receive any postoperative treatment. Because of the variation on treatment and lack of study assessed, it is difficult to say whether the prognostic effect of c-Met is associated with clinical treatment or not based on available studies. Further studies are still warranted to better clarify this association.

To our knowledge, this is the first meta-analysis that strongly suggests high c-Met status represent adverse prognostic biomarker for overall survival in GC patients. Patients with tumors that harbour high c-Met expression are more likely to have a worse OS, and this prognostic effect was independent to disease stage. However, large studies using standardized unbiased methods are still required before c-Met testing can move toward routine clinical application as a prognostic tool.

Collectively, this study’s overall findings support the hypothesis that c-Met expression level is associated with overall survival in GC patients. Future studies should try to block HGF/c-Met pathway so as to prolong the overall survival of GC patients, and the prognosis of patients harbouring high c-Met expression would be changed with more and more clinical trials evaluating targeted agents against HGF or c-Met in GC patients.

## Supporting Information

Table S1 **Clinical treatment of studies reporting c-Met expression and outcomes in gastric cancer patients.**
(DOC)

Figure S1 **Forest plot showing the meta-analysis of hazard ratio estimates for OS in postoperative and no postoperative treatment subgroup.**
(TIF)

Checklist S1 **The section that contains each item in PRISMA Checklist.**
(DOC)

## References

[1] Jemal A, Bray F, Center MM, Ferlay J, Ward E et al. (2011) Global cancer statistics. CA Cancer J Clin 61(2): 69-90. doi:10.3322/caac.20107. PubMed: 21296855. 21296855

[2] Ajani JA (2008) Gastroesophageal cancers: Progress and problems. J Natl Compr Canc Netw 6(9): 813-814. PubMed: 18998257. 18998257

[3] Goscinski MA, Larsen SG, Warloe T, Stoldt S, Nesland JM et al. (2009) Adenocarcinomas on the rise–does it influence survival from oesophageal cancer? Scand J Surg 98(4): 214-220. PubMed: 20218417. 20218417 10.1177/145749690909800404

[4] Huang TJ, Wang JY, Lin SR, Lian ST, Hsieh JS (2001) Overexpression of the c-met protooncogene in human gastric carcinoma: Correlation to clinical features. Acta Oncol 40(5): 638-643. doi:10.1080/028418601750444204. PubMed: 11669338. 11669338

[5] Wu CW, Li AF, Chi CW, Chung WW, Liu TY et al. (1998) Hepatocyte growth factor and Met/HGF receptors in patients with gastric adenocarcinoma. Oncol Rep 5(4): 817-822. PubMed: 9625824. 9625824 10.3892/or.5.4.817

[6] Inoue T, Kataoka H, Goto K, Nagaike K, Igami K et al. (2004) Activation of c-Met (hepatocyte growth factor receptor) in human gastric cancer tissue. Cancer Sci 95(10): 803-808. doi:10.1111/j.1349-7006.2004.tb02185.x. PubMed: 15504247. 15504247 PMC11158965

[7] Nakajima M, Sawada H, Yamada Y, Watanabe A, Tatsumi M et al. (1999) The prognostic significance of amplification and overexpression of c-met and c-erb B-2 in human gastric carcinomas. Cancer 85(9): 1894-1902. doi:10.1002/(SICI)1097-0142(19990501)85:9<1894::AID-CNCR3>3.0.CO;2-J. PubMed: 10223227. 10223227 10.1002/(sici)1097-0142(19990501)85:9<1894::aid-cncr3>3.0.co;2-j

[8] Lennerz JK, Kwak EL, Ackerman A, Michael M, Fox SB et al. (2011) MET amplification identifies a small and aggressive subgroup of esophagogastric adenocarcinoma with evidence of responsiveness to crizotinib. J of clininical Oncology 29(36): 4803-4810. doi:10.1200/JCO.2011.35.4928. PubMed: 22042947. PMC3255989 22042947

[9] Kuniyasu H, Yasui W, Kitadai Y, Yokozaki H, Ito H et al. (1992) Frequent amplification of the c-met gene in scirrhous type stomach cancer. Biochem Biophys Res Commun 189(1): 227-232. doi:10.1016/0006-291X(92)91548-5. PubMed: 1333188. 1333188

[10] Amemiya H, Kono K, Itakura J, Tang RF, Takahashi A et al. (2002) c-Met expression in gastric cancer with liver metastasis. Oncology 63(3): 286-296. doi:10.1159/000065477. PubMed: 12381909. 12381909

[11] Retterspitz MF, Mönig SP, Schreckenberg S, Schneider PM, Hölscher AH et al. (2010) Expression of {beta}-catenin, MUC1 and c-met in diffuse-type gastric carcinomas: correlations with tumour progression and prognosis. Anticancer Res 30(11): 4635-4641. PubMed: 21115917. 21115917

[12] Tang Z, Zhao M, Ji J, Yang G, Hu F et al. (2004) Overexpression of gastrin and c-met protein involved in human gastric carcinomas and intestinal metaplasia. Oncol Rep 11(2): 333-339. PubMed: 14719064. 14719064

[13] Conrotto P, Valdembri D, Corso S, Serini G, Tamagnone L et al. (2005) Sema4D induces angiogenesis through Met recruitment by Plexin B1. Blood 105(11): 4321-4329. doi:10.1182/blood-2004-07-2885. PubMed: 15632204. 15632204

[14] Yi S, Tsao MS (2000) Activation of hepatocyte growth factor-met autocrine loop enhances tumorigenicity in a human lung adenocarcinoma cell line. Neoplasia 2(3): 226-234. PubMed: 10935508. 10935508 10.1038/sj.neo.7900080 PMC1507572

[15] Kelly S, Oliner, Tang Rui, Anderson Abraham, Lan Yun, Timothy Iveson et al. (2012) Evaluation of MET pathway biomarkers in a phase II study of rilotumumab (R, AMG 102) or placebo (P) in combination with epirubicin, cisplatin, and capecitabine (ECX) in patients (pts) with locally advanced or metastatic gastric (G) or esophagogastric junction (EGJ) cancer. J Clin Oncol 30(suppl; abstr: 4005).

[16] Parmar MKB, Torri V, Stewart L (1998) Extracting summary statistics to perform Meta-analyses of the published literature for survival endpoints. Statist Med 17(24): 2815-2834. doi:10.1002/(SICI)1097-0258(19981230)17:24. PubMed: 9921604. 9921604

[17] Wu CW, Li AF, Chi CW, Chung WW, Liu TY et al. (1998) Hepatocyte growth factor and Met/HGF receptors in patients with gastric adenocarcinoma. Oncol Rep 5(4): 817-822. PubMed: 9625824. 9625824 10.3892/or.5.4.817

[18] Yonemura Y, Nojima N, Kaji M, Kawamura T, Fushida S et al. (1997) E-cadherin and c-met expression as a prognostic factor in gastric cancer. Oncol Rep 4(4): 743-748. PubMed: 21590132. 21590132 10.3892/or.4.4.743

[19] Shi J, Yao D, Liu W, Wang N, Lv H et al. (2012). nt J Molecular Sciences Frequent Gene Amplif Predicts Poor Prognosis Gastric Cancer 13(4): 4714-4726 10.3390/ijms13044714 PMC3344242 22606006

[20] Graziano F, Galluccio N, Lorenzini P, Ruzzo A, Canestrari E et al. (2011) Genetic activation of the MET pathway and prognosis of patients with high-risk, radically resected gastric cancer. J Clin Oncol 29(36): 4789-4795. doi:10.1200/JCO.2011.36.7706. PubMed: 22042954. 22042954

[21] Zhao J, Zhang X, Xin Y (2011) Up-regulated expression of Ezrin and c-Met proteins are related to the metastasis and prognosis of gastric carcinomas. Histol Histopathol 26(9): 1111-1120. PubMed: 21751142. 21751142 10.14670/HH-26.1111

[22] Lee J, Seo JW, Jun HJ, Ki CS, Park SH et al. (2011) Impact of MET amplification on gastric cancer: possible roles as a novel prognostic marker and a potential therapeutic target. Oncol Rep 25(6): 1517-1524. PubMed: 21424128. 21424128 10.3892/or.2011.1219

[23] Drebber U, Baldus SE, Nolden B, Grass G, Bollschweiler E et al. (2008) The overexpression of c-met as a prognostic indicator for gastric carcinoma compared to p53 and p21 nuclear accumulation. Oncol Rep 19(6): 1477-1483. PubMed: 18497953. 18497953

[24] Han SU, Lee HY, Lee JH, Kim WH, Nam H et al. (2005) Modulation of E-cadherin by hepatocyte growth factor induces aggressiveness of gastric carcinoma. Ann Surg 242(5): 676-683. doi:10.1097/01.sla.0000186171.85804.fe. PubMed: 16244541. 16244541 PMC1409863

[25] Kubicka S, Claas C, Staab S, Kühnel F, Zender L et al. (2002) p53 mutation pattern and expression of c-erbB2 and c-met in gastric cancer: relation to histological subtypes, Helicobacter pylori infection, and prognosis. Dig Dis Sci 47(1): 114-121. doi:10.1023/A:1013275706401. PubMed: 11837710. 11837710

[26] Tsugawa K, Yonemura Y, Hirono Y, Fushida S, Kaji M et al. (1998) Amplification of the c-met, c-erbB-2 and epidermal growth factor receptor gene in human gastric cancers: correlation to clinical features. Oncology 55(5): 475-481. doi:10.1159/000011898. PubMed: 9732228. 9732228

[27] Taniguchi K, Yonemura Y, Nojima N, Hirono Y, Fushida S et al. (1998) The relation between the growth patterns of gastric carcinoma and the expression of hepatocyte growth factor receptor (c-met), autocrine motility factor receptor, and urokinase-type plasminogen activator receptor. Cancer 82(11): 2112-2122. doi:10.1002/(SICI)1097-0142(19980601)82:11. PubMed: 9610690. 9610690

[28] Li Y, Chen CQ, He YL, Cai SR, Yang DJ et al. (2012) Abnormal Expression of E-Cadherin in Tumor Cells is Associated With Poor Prognosis of Gastric Carcinoma. J Surg Oncol 106(3): 304-310. doi:10.1002/jso.23008. PubMed: 22231933. 22231933

[29] Lee HE, Kim MA, Lee HS, Jung EJ, Yang HK et al. (2012) MET in gastric carcinomas: comparison between protein expression and gene copy number and impact on clinical outcome. Br J Cancer 107(2): 325-333. doi:10.1038/bjc.2012.237. PubMed: 22644302. 22644302 PMC3394975

[30] Catenacci DV, Cervantes G, Yala S, Nelson EA, El-Hashani E et al. (2011) RON (MST1R) is a novel prognostic marker and therapeutic target for gastroesophageal adenocarcinoma. Cancer Biol&thrapy 12(1): 9-46. doi:10.4161/cbt.12.1.15747. PubMed: 21543897. PMC3149873 21543897

[31] Toiyama Y, Yasuda H, Saigusa S, Matushita K, Fujikawa H et al. (2012) Co-expression of hepatocyte growth factor and c-Met predicts peritoneal dissemination established by autocrine hepatocyte growth factor/c-Met signaling in gastric cancer. Int J Cancer 130(12): 2912-2921. doi:10.1002/ijc.26330. PubMed: 21796631. 21796631

[32] Hofmann M, Stoss O, Shi D, Büttner R, van de Vijver M et al. (2008) Assessment of a HER2 scoring system for gastric cancer: results from a validation study. Histopathology Jun 52(7): 797-805. doi:10.1111/j.1365-2559.2008.03028.x. PubMed: 18422971. 18422971

[33] Gill S, Shah A, Le N, Cook EF, Yoshida EM (2003) Asian ethnicity-related differences in gastric cancer presentation and outcome among patients treated at a canadian cancer center. J Clin Oncol 21(11): 2070-2076. doi:10.1200/JCO.2003.11.054. PubMed: 12775731. 12775731

[34] Kim J, Sun CL, Mailey B, Prendergast C, Artinyan A (2010) Race and ethnicity correlate with survival in patients with gastric adenocarcinoma. Ann Oncol 21(1): 152-160. doi:10.1093/annonc/mdp290. PubMed: 19622590. 19622590

