# Supplementary material for: c-Met as a Prognostic Marker in Gastric Cancer: A Systematic Review and Meta-Analysis
Source: PLoS One. 2013 Nov 4;8(11):e79137. doi: 10.1371/journal.pone.0079137 (PMC3817069; doi:10.1371/journal.pone.0079137)
Supplement: Table S1 — Clinical treatment of studies reporting c-Met expression and outcomes in gastric cancer patients. (DOC) [file pone.0079137.s001.doc]

Table S1. Clinical treatment of studies reporting

c-Met expression and outcomes in gastric cancer patients

| Study ID | Disease stage | surgery | Postoperative treatment | Palliative treatment |
| --- | --- | --- | --- | --- |
| Toiyama 2011 | I-IV | Curative or noncurative | NS^a^ | NS |
| Catenacci 2011 | I-IV | Curative or noncurative | NS | NS |
| Catenacci 2011 | I-IV | Curative or noncurative | NS | NS |
| Lee 2012 | I-IV | Curative or noncurative | no or fluorouracil-based regimen | NS |
| Li 2012 | I-IV | Curative or noncurative | modified FOLFOX6 regimen | NS |
| Taniguchi 1998 | I-IV | Curative or noncurative | no or fluorouracil-based regimen | NS |
| Tsugawa 1998 | I-IV | Curative or noncurative | NS | NS |
| Nakajima 1999 | I-III | curative | no or fluorouracil-based regimen | NS |
| Huang 2001 | I-IV | Curative or noncurative | NS | NS |
| KUBICKA 2002 | I-III | curative | NS | NS |
| Han 2005 | I-IV | Curative or noncurative | NS | NS |
| DREBBER 2008 | I-IV | Curative or noncurative | NS | NS |
| Lee 2011 | I-IV | Curative | fluorouracil-based chemotherapy in combination with radiotherapy | NS |
| Zhao 2011 | I-III | curative | none | NS |
| Graziano 2011 | II-III | curative | NS | All patients who experienced relapse underwent fluorouracil-based chemotherapy |
| Shi 2012 | I-IV | Curative or noncurative | NS | NS |

^a^: NS=not shown
