# Supplementary figures and images for: c-Met as a Prognostic Marker in Gastric Cancer: A Systematic Review and Meta-Analysis
Source: PLoS One. 2013 Nov 4;8(11):e79137. doi: 10.1371/journal.pone.0079137 (PMC3817069; doi:10.1371/journal.pone.0079137)

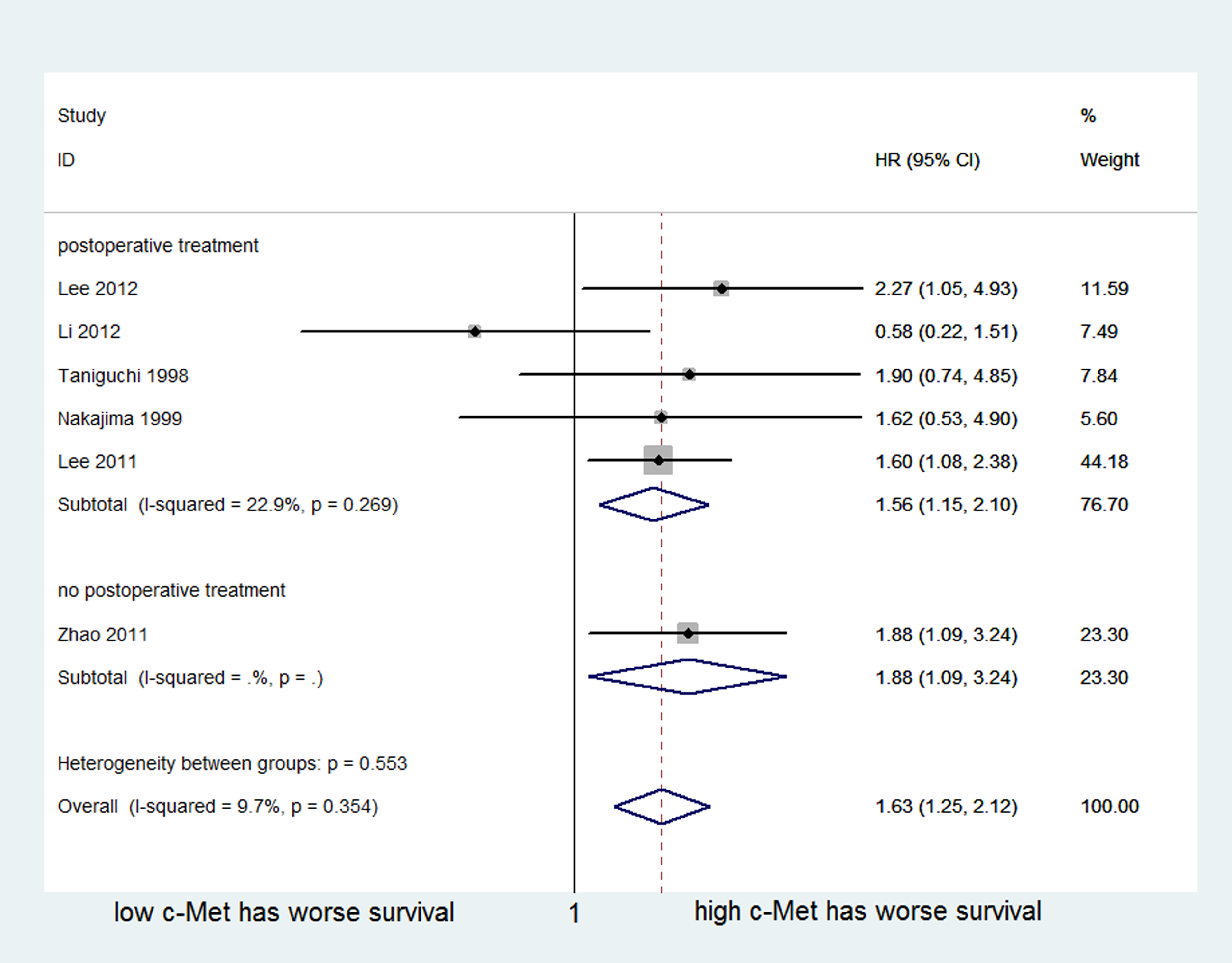

Supplement: Figure S1 — Forest plot showing the meta-analysis of hazard ratio estimates for OS in postoperative and no postoperative treatment subgroup. (TIF) [file pone.0079137.s002.tif]
